# Supplementary material for: Absence makes the heart grow fonder: social compensation when failure to interact risks weakening a relationship
Source: EPJ Data Sci. 2017 Jan 5;6(1):1. doi: 10.1140/epjds/s13688-016-0097-x (PMC7175705; doi:10.1140/epjds/s13688-016-0097-x)
Supplement: Supplementary file 1 — Supplementary material. (pdf) [file 13688_2016_97_MOESM1_ESM.pdf]

SUPPLEMENTARY MATERIAL (SM)

Absence makes the heart grow fonder: social compensation when failure to interact risks weakening a relationship

Kunal Bhattacharya,[1,*] Asim Ghosh,[1] Daniel Monsivais,[1] Robin Dunbar,[2,1] and Kimmo Kaski[1,2]

[1]*Department of Computer Science, Aalto University School of Science, P.O. Box 15400, FI-00076 AALTO, Finland*

[2]*Department of Experimental Psychology, University of Oxford, South Parks Rd, Oxford, OX1 3UD, United Kingdom*

[*] Corresponding author; kunal.bhattacharya@aalto.fi

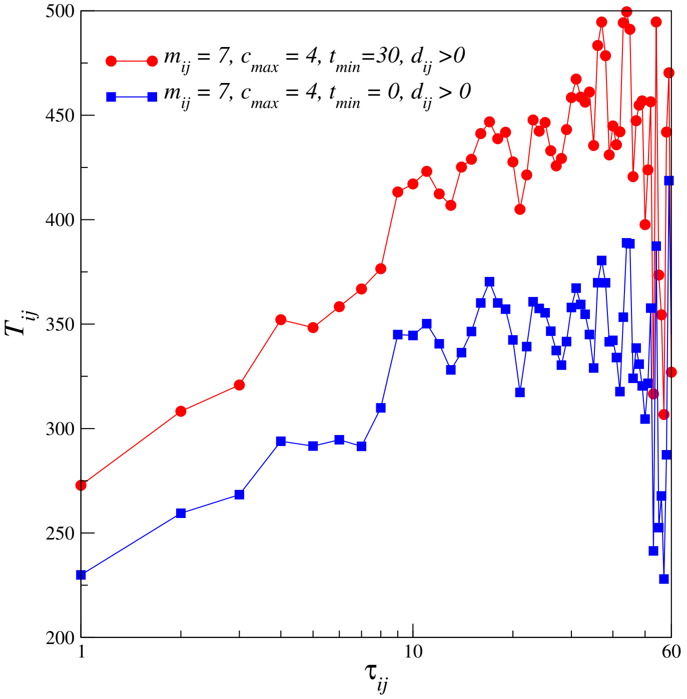

FIG. S1. **Linear binnining of data for different thresholds (t_{min}).** Duration of succeeding call (T_{ij}) as a function of the gap (τ_{ij}) for pairs belonging to the set $\mathcal{S}$ with the individuals aged between 25 and 45. Two different thresholds are used for the total aggregated duration in the 7 month period – $t_{min} = 30$ minutes (red circles) and $t_{min} = 0$ (no threshold) (blue squares). Linear binning is used for this plot unlike the log-binning used in the main text for reducing the fluctuation. The curves show a periodic drop in the call duration on multiples in 7 days. This behaviour is related to the presence of peaks in the distribution of the gaps (τ_{ij}). As such larger number of calls on the days at 'weekends' result in a drop in the average duration of a call on those days. The equation we have used for regression primarily takes into account the dependence $T_{ij} \propto \log \tau_{ij}$. We have not taken into account the periodicity present in the data.

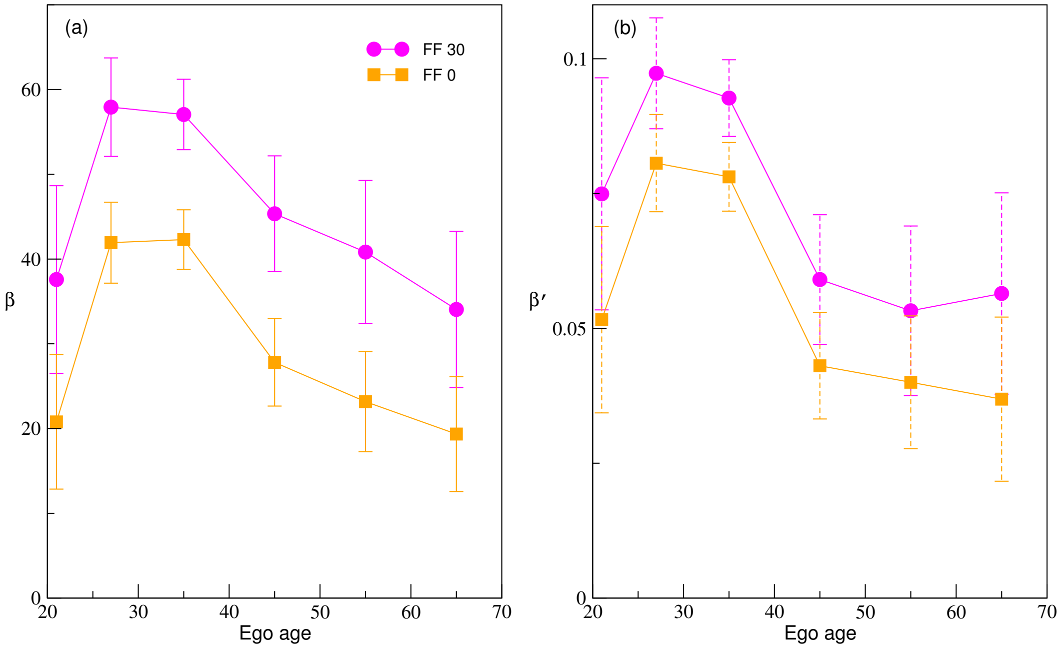

FIG. S2. **Values of β and β' for different thresholds (t_{min}).** Comparison of the slopes of the regression fit (main text), β (a) and β' (b), for values of t_{min} equal to 30 minutes (circles) and zero (implying no threshold) (squares). For clarity only the values corresponding to female-female (FF) pairs chosen from set $\mathcal{S}$ are shown. The values of β' show that even without a finite threshold on the calling duration, the values are different from zero.

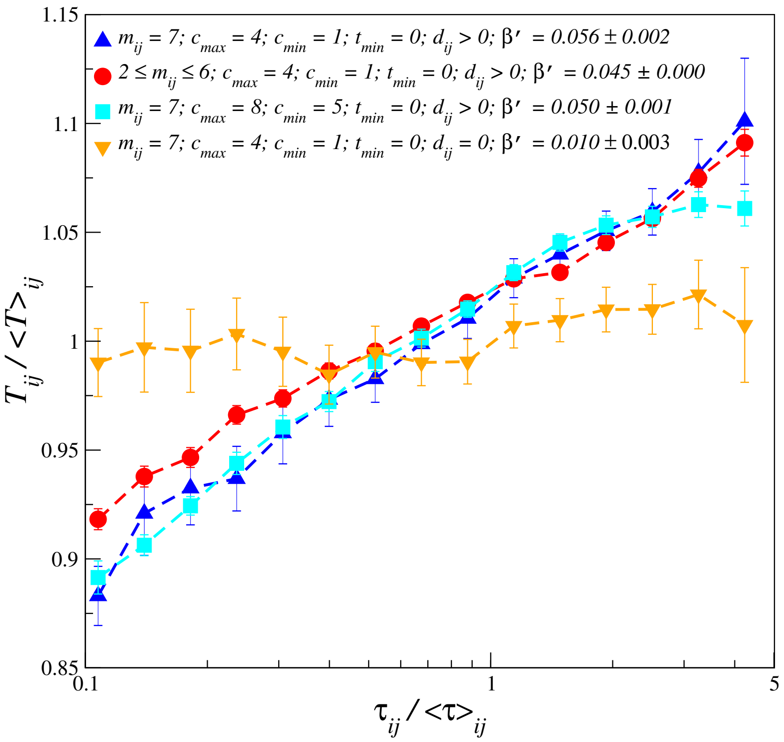

FIG. S3. **Effect of using different filtering parameters.** The figure illustrates the dependence of the scaled durations, $T_{ij}/\langle T\rangle_{ij}$ on scaled gaps $\tau_{ij}/\langle\tau\rangle_{ij}$ when the pairs are chosen using one or more conditions (m_{ij}, c_{max}, t_{min} and d_{ij}) which are different from those that are used to construct $\mathcal{S}$. (We use an additional parameter c_{min} which denotes the minimum number of calls between a pair in a any month. For $\mathcal{S}$, at least one call every month ensures $c_{min} = 1$). The pairs are sampled irrespective of the age and gender of the individuals. The curve with blue up-triangles with no lower limit is set on the aggregated call duration in the 7 month period ($t_{min} = 0$, rest of the parameters being the same as in $\mathcal{S}$). The curve with red circles shows the behaviour when the condition $m_{ij} = 7$ is relaxed. The pairs chosen are those that participate in calls in different months numbering from a minimum of 2 to a maximum of 6. The cyan squares show the case when the minimum number of calls in each of the 7 months is set to 5 and the maximum number is 8, in contrast to the maximum being 4 in $\mathcal{S}$. The orange down-triangles illustrate the case when pairs having the same most common locations are chosen. This curve is almost flat allowing for fluctuations.

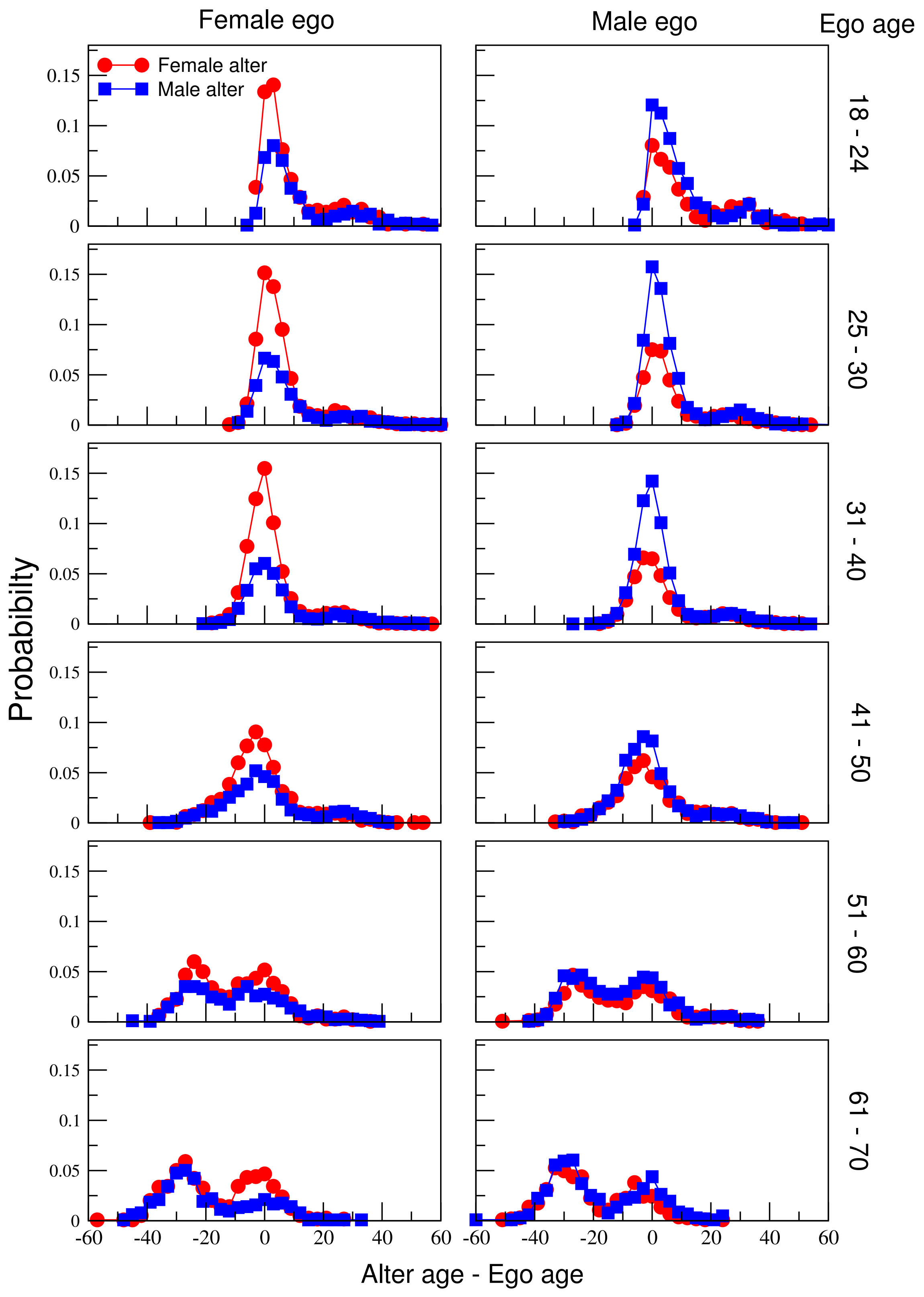

FIG. S4. **Gender of individuals in the chosen pairs.** Distribution of (alter age−ego age) for female and male egos in different age cohorts with alters belong to the set $\mathcal{S}$.

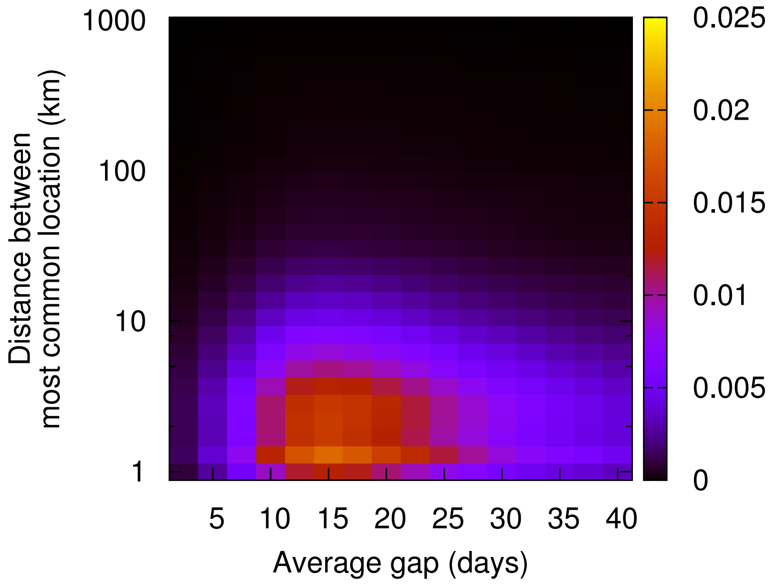

FIG. S5. **Distribution of inter-call gap and geographical separation of individuals making up pairs.** Joint probabilty density function of average gap between pairs ($\langle\tau\rangle_{ij}$) and the distance between their most common location (d_{ij}). Pairs are chosen irrespective of age and gender.

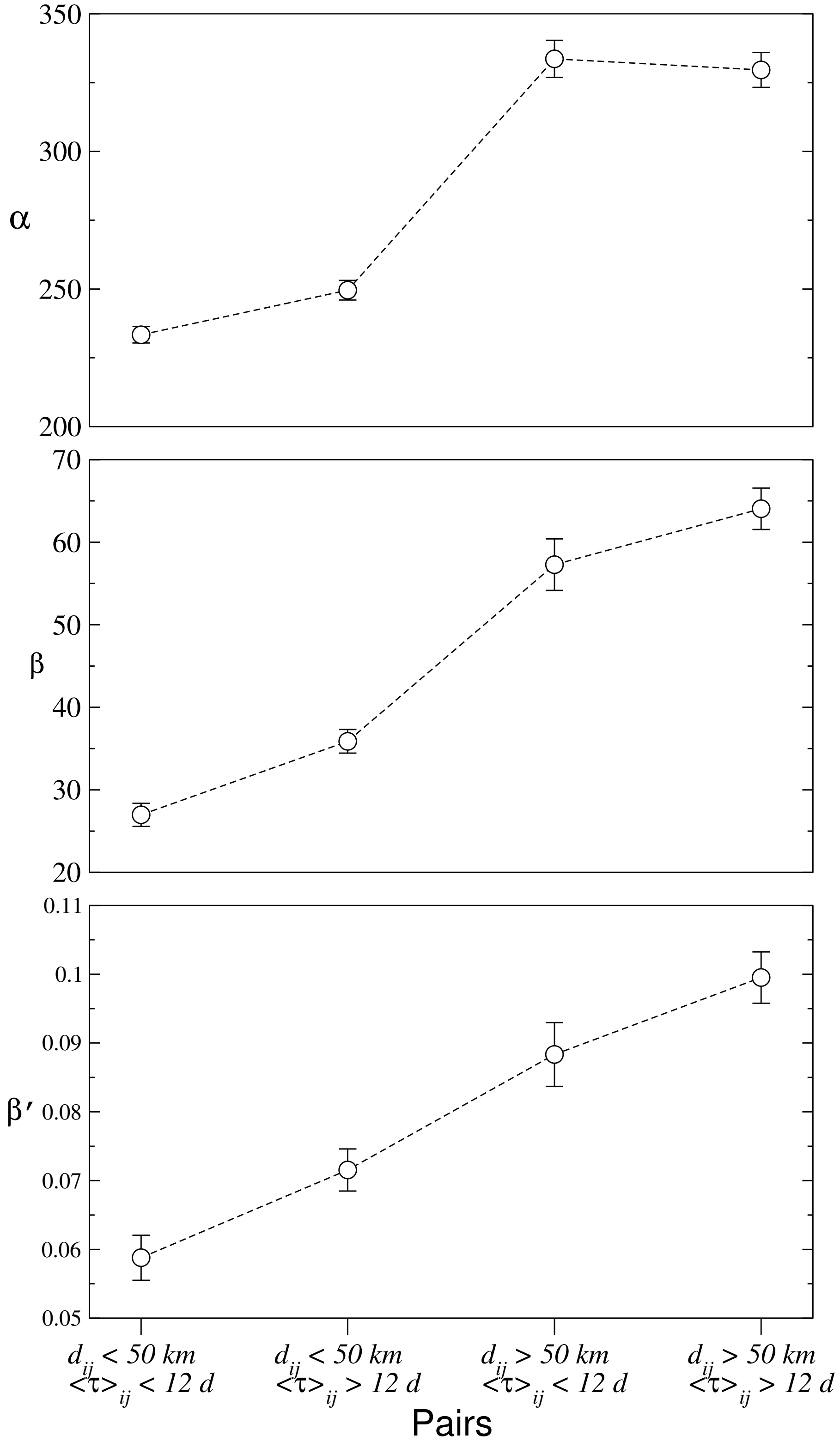

FIG. S6. **Regression coefficients irrespective of the gender of individuals in the pairs.** Coefficients α, β and β' corresponding four different categories based on distance between most common location (d_{ij}) and average gap ($\langle\tau\rangle_{ij}$). Pairs are chosen irrespective of age and gender. The dashed line is a guide to the eye. (Also see Fig. 5 in the main text).

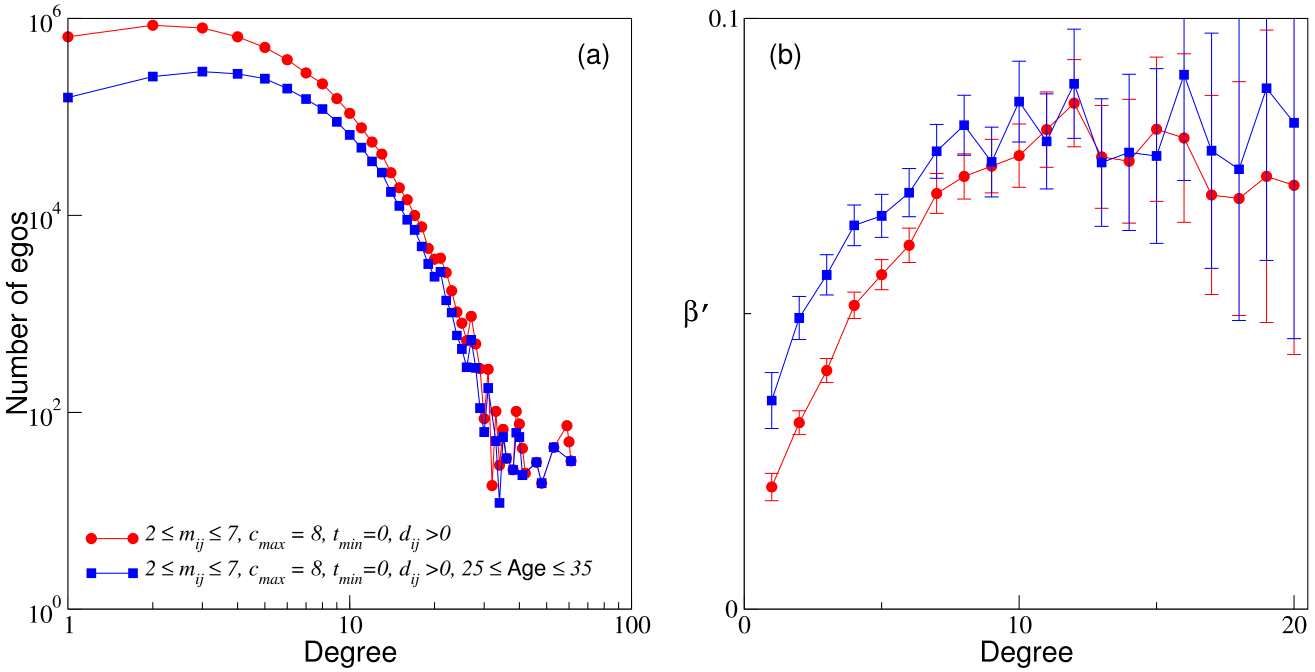

FIG. S7. **Dependence on degree.** (a) The distribution of degree of egos. (b) Coefficient β' as a function of degree (β' is the strength of the dependence of the duration of the succeeding call on the logarithm of the inter-call gap when the variables are scaled by their respective averages). The different symbols represent the criteria for choosing the sets of ego-alter pairs, and are indicated in the legend of (a). The egos in the set (represented by squares) were chosen such that their ages belonged to the range $[25, 35]$. We restrict our analysis to a maximum degree of 20. For the larger values of degree, the lesser number of samples weakens the statistical significance of the results.
